# A scoring system derived from electronic health records to identify patients at high risk for noninvasive ventilation failure

**DOI:** 10.1186/s12890-021-01421-w

**Published:** 2021-02-05

**Authors:** Mihaela S. Stefan, Aruna Priya, Penelope S. Pekow, Jay S. Steingrub, Nicholas S. Hill, Tara Lagu, Karthik Raghunathan, Anusha G. Bhat, Peter K. Lindenauer

**Affiliations:** 1 Institute for Healthcare Delivery and Population Science, University of Massachusetts Medical School - Baystate, Springfield, MA USA; 2 Department of Medicine, University of Massachusetts Medical School - Baystate, Springfield, MA USA; 3 School of Public Health and Health Sciences, University of Massachusetts, Amherst, MA USA; 4 Division of Pulmonary and Critical Care, Department of Medicine, University of Massachusetts Medical School - Baystate, Springfield, MA USA; 5 Division of Pulmonary and Critical Care, Tufts University School of Medicine, Boston, MA USA; 6 Division of Veterans Affairs, Department of Anesthesiology, Duke University Medical Center, Durham, NC USA; 7 Department of Quantitative Health Sciences, University of Massachusetts Medical School, Worcester, MA USA

**Keywords:** Intubation, noninvasive ventilation failure, Predictive score, Acute respiratory failure, Mechanical ventilation

## Abstract

**Objective:**

To develop and validate a clinical risk prediction score for noninvasive ventilation (NIV) failure defined as intubation after a trial of NIV in non-surgical patients.

**Design:**

Retrospective cohort study of a multihospital electronic health record database.

**Patients:**

Non-surgical adult patients receiving NIV as the first method of ventilation within two days of hospitalization.

**Measurement:**

Primary outcome was intubation after a trial of NIV. We used a non-random split of the cohort based on year of admission for model development and validation. We included subjects admitted in years 2010–2014 to develop a risk prediction model and built a parsimonious risk scoring model using multivariable logistic regression. We validated the model in the cohort of subjects hospitalized in 2015 and 2016.

**Main results:**

Of all the 47,749 patients started on NIV, 11.7% were intubated. Compared with NIV success, those who were intubated had worse mortality (25.2% vs. 8.9%). Strongest independent predictors for intubation were organ failure, principal diagnosis group (substance abuse/psychosis, neurological conditions, pneumonia, and sepsis), use of invasive ventilation in the prior year, low body mass index, and tachypnea. The c-statistic was 0.81, 0.80 and 0.81 respectively, in the derivation, validation and full cohorts. We constructed three risk categories of the scoring system built on the full cohort; the median and interquartile range of risk of intubation was: 2.3% [1.9%–2.8%] for low risk group; 9.3% [6.3%–13.5%] for intermediate risk category; and 35.7% [31.0%–45.8%] for high risk category.

**Conclusions:**

In patients started on NIV, we found that in addition to factors known to be associated with intubation, neurological, substance abuse, or psychiatric diagnoses were highly predictive for intubation. The prognostic score that we have developed may provide quantitative guidance for decision-making in patients who are started on NIV.

## Introduction

Noninvasive ventilation (NIV) plays a key role in the treatment of acute respiratory failure (ARF) and its use is supported by multiple randomized controlled trials [1–4]. The evidence of benefit is strong for patients with acute hypercapnic respiratory failure [1, 5] and cardiogenic pulmonary edema [6, 7], while consistent benefit in other conditions such as acute hypoxemic respiratory failure was not found [8–10]. Even so, the use of NIV has dramatically increased in the last two decades in the US for all diagnoses regardless of supporting evidence [11–14].

Therapy with NIV is considered successful if endotracheal intubation is avoided. Conversely, the term NIV failure is used when a patient initially treated with NIV requires invasive mechanical ventilation (IMV) or dies without being intubated. NIV failure rates range from 5 to 50% and patients who are intubated have an increased risk of death compared to those treated with IMV from the outset [9, 11, 15–17]. Determining which patients are appropriate for NIV therapy is a complex decision that requires assessment of an individual’s chances of failure and/or survival; improper patient selection is a main reason for poor outcomes [18–20]. Prior studies have identified several risk factors associated with NIV failure including coexistent pneumonia, tachypnea, hypotension, severe acidemia, higher severity of illness score, or failure to improve in one hour. However, most of these studies were small, were developed in cohorts from randomized trials, or were geared towards specific diagnoses [15, 21–24].

A simple risk score developed in a real-world cohort to identify patients’ risk for NIV failure may support clinical decision for initiation of NIV and trigger goals of care discussions at the time of NIV initiation. It may also help with decisions regarding monitoring; patients at low risk of failure could be potentially admitted in a step-down unit, whereas those at high risk could benefit from admission to an intensive care unit. Therefore, using data from a large multihospital electronic health record database that contains vitals and laboratory results, we sought to develop a clinical risk score for NIV failure defined as intubation after a trial of NIV based on information routinely available to clinicians at the time of NIV initiation.

## Materials and methods

### Design, data source, and population

We conducted a retrospective cohort study using an electronic health record dataset, Cerner HealthFacts from January 2010 to July 2016. Health Facts contains data on patient demographics, diagnoses and procedures and detailed, time-stamped, clinical, pharmacy and laboratory results. For this analysis, we included 127 hospitals that contributed data to all domains (laboratory, pharmacy, vitals, and administrative data). We included non-surgical patients 18 years or older with NIV initiated in the first two days of hospitalization. We excluded patients receiving palliative care or hospice, patients with obstructive sleep apnea (we could not ascertain if NIV was used for OSA or for acute respiratory failure), and patients transferred to or from another facility. We used ICD-9 CM and ICD-10 CM procedure codes to identify NIV treatment; prior studies have shown that these procedure codes have sensitivity of 86.5% and specificity of 91.5% [25].

### Candidate risk factors

Potential candidate variables were identified based on a review of the literature and clinical relevance [15, 16, 21–24]. Patients were grouped into the following 11 major categories based on the evidence for NIV use and size of the cohort: (1) congestive heart failure (CHF), (2) acute myocardial infarction (AMI) (3) chronic obstructive pulmonary disease (COPD), (4) asthma, (5) pneumonia, (6) sepsis, (7) stroke, (8) neurological non-stroke diagnosis, (9) substance abuse, (10) psychiatric diagnoses, (11) others. To assess severity of illness we used the following variables: number of hospitalizations and NIV or IMV use in the year prior to the index admission; vasopressor use within first two days of hospitalization and organ failure (acute respiratory failure was not counted) [26, 27]. We also included the following comorbidities known to be associated with NIV failure: chronic pulmonary disease, neurological disorders, psychiatric disorders, substance abuse, obesity, and weight loss. Vitals and laboratory variables known to be predictive for NIV failure such as respiratory rate, heart rate, blood pressure or bicarbonate were grouped into categories based on the Laboratory Acute Physiology Score (LAPS), which uses the results of laboratory testing around the time of admission to quantify the risk of inpatient mortality [28, 29]*.* For example, respiratory rate in our model was categorized as ≤ 29versus  ≥ 30 with points assigned for patients with high respiratory rate [28]. We grouped missing values as separate category for factors that contained them and included in all analyses.

### Outcomes

The primary outcome was intubation following a trial of NIV.

Our secondary outcome was NIV failure defined as intubation or death.

### Statistical analysis

We computed summary statistics to characterize the cohort and calculated standardized mean differences to compare groups with and without the outcome of interest; a difference of > 10% is deemed significant [30].

Derivation and validation data sets: we split the cohort non-randomly based on year of admission with patients admitted in years 2010–2014 for model derivation and patients admitted in years 2015–2016 for validation. We first computed Spearman’s rank correlations between factors to check for any collinearity between predictors We developed a series of multivariable logistic regression models to predict intubation in the derivation cohort. We initially included all the candidate risk factors, and then removed those that did not add significantly to the model [31]. We used a backward selection process wherein we selected factors based on their contribution to the model via Type 3 sums of squares. Further, to increase the likelihood that the model will be used in real-time for risk stratification purpose, we reduced the number of variables: first, we selected those with the strongest predictive ability; second, we combined candidate factors that were clinically comparable and had a similar magnitude of effect. Factors that had greater contribution to the model were selected with a cut-off at the top 10 variables. The final model was fit and checked for model performance using the c-statistics and compared it against the full model performance. Discrimination was evaluated by the area under the receiver operating characteristic (ROC) curve [32] and calibration was measured by review of the calibration plots. Parameter estimates obtained from the derivation cohort were then used to compute individual intubation risk in the validation cohort of patients admitted in year 2015 and 2016. Models were assessed for possible overfitting using the least absolute shrinkage and selection operator (LASSO) method [33]. To assess the robustness of the model we used a fivefold cross-validation technique [34]. We divided the full cohort into 5 segments (“folds”) and then refit the model that we developed in the derivation cohort in 4 of the “folds” (80% data) and validated it on the remaining fold (20% data). This was performed a total of 5 times, leaving out a different “fold” each time; we then computed c-statistics as a measure of assessment of model fit for each validation “fold”. Finally, we fit the model in full cohort and then developed a point-scoring system for intubation using a regression coefficient-based scoring method [35]. The total risk score was calculated by adding each component, and intubation rates were determined for the various scores. We then computed the probability of intubation at different cut-points for the total score and constructed 3 categories of intubation risk (low, medium and high). We employed similar a analytic strategy for the NIV failure outcome defined as intubation or death.

All analyses were conducted using SAS statistical software (Version 9.4; SAS Institute Inc, Cary, NC) and Stata statistical software (Version 15; STATA Corp, College Station, Texas).

The study has been performed in accordance with the Declaration of Helsinki and has been approved by the Baystate Institutional Review Board. Informed consent was not required as this was a retrospective study of de-identified data.

## Results

**Fig. 1:**
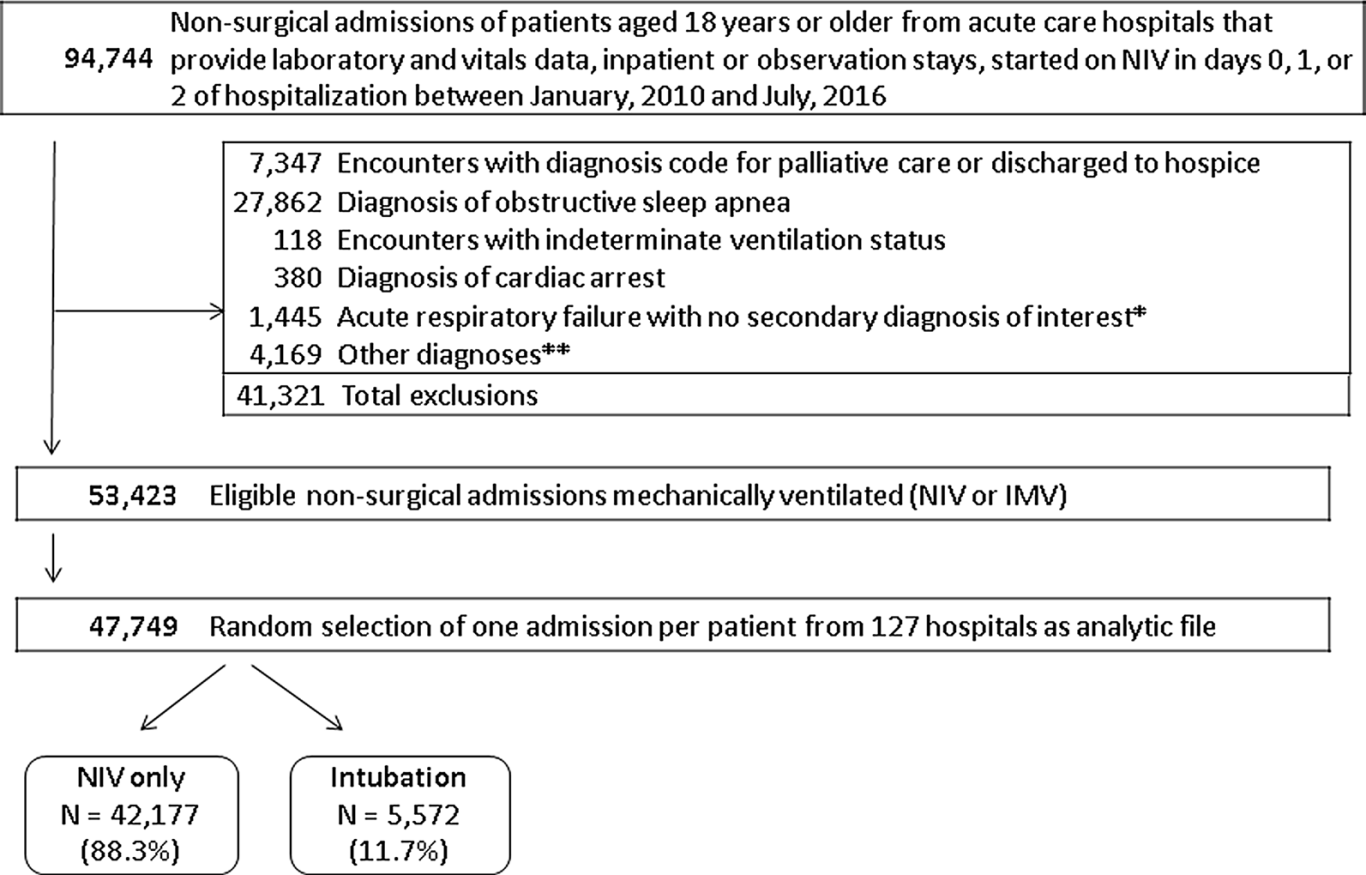
Patient selection flow chart

There were 94,744 hospitalizations of non-surgical patients with NIV initiated within the first 2 days of admission at 127 hospitals. After exclusion criteria were applied (Fig. 1), our full cohort consisted of 47,749 patients.

### Cohort characteristics

47,749 patients were included in our analysis; median age was of 65 years, 48.2% were female and 74.8% were of white race. The most common principal diagnoses were sepsis or pneumonia (23.5%), AMI (22.7%), and COPD/asthma (17.5%); 40% of patients had one or more prior admissions and 11.5% had received NIV in the prior year. About thirty-seven percent of the patients had one or more organ failures (in addition to acute respiratory failure), 16.4% were treated with vasopressors. Among the 43,277 patients initiated on NIV by day 1, 75.5% were started in the emergency department. Summary statistics of demographics, comorbidities, and presenting features are shown in Additional file 1: Table E1. In-hospital mortality was 10.8% and the median length of stay was 5 days (IQR: 3–9). Overall, 5,572 (11.7%) patients were intubated and 1,402 (25.2%) of those who intubated died.

### Characteristics of patients who were intubated

Compared to patients who were not intubated, patients who were intubated were younger (median age 62 vs. 66 years), more likely to be of Black race (22.0% vs. 15.4%), and more likely to have been treated with IMV in the prior year (6.8% vs. 2.7%). Those who were intuabted were sicker, with higher rates of comorbidities including neurological disorders (21.8% vs. 9.8%), liver disease (7.3% vs. 3.1%), and psychiatric disorders (20.4% vs. 11.8%); they had higher use of vasopressors (25.0% vs. 16.6%); were more likely to have one or more organ failures (67.3% vs. 29.5%) or comorbid pneumonia (33.0% vs. 20.0%); and higher admission LAPS score (median score 50 vs. 40) (Additional file 1: Table E1). Compared with those in whom NIV was successful, those who were intubated had higher mortality (25.2% vs. 8.9%; p-value < 0.001) and longer length of hospital stay [median (IQR): 8 (3–16) vs. 5 (2–8); p-value < 0.001].

Rates of intubation or death after intubation varied dramatically by condition; for example, patients with substance abuse or a psychiatric diagnosis had the highest intubationrate of 29.3% but a low mortality after intubation of 7.3%, while patients with AMI had the lowest intubation rate of 3.9% but a high mortality among those intubated of 34.2%; patients with stroke had both high intubation rate (21.0%) and mortality after intubation (35.7%). (Table 1).

**Table 1:** Rates of overall death, intubation, no intubation, death with and without intubation in patients treated with noninvasive ventilation

Diagnosis	Entire cohort	Death overall	Intubation	Death after intubation	No. intubation	Death without intubation	p-value*
	N (column %)	Row %	Row %	Row %	Row %	Row %	
Total number of patients	47,749 (100)	5,150 (10.8)	5,572 (11.7)	1,402 (25.2)	42,177 (88.3)	3,748 (8.9)	
COPD/ASTHMA	8346 (17.5)	470 (5.6)	941 (11.3)	155 (16.5)	7405 (88.7)	315 (4.3)	< 0.001
CHF	4896 (10.3)	336 (6.9)	351 (7.2)	90 (25.6)	4545 (92.8)	246 (5.4)	< 0.001
AMI	10,820 (22.7)	663 (6.1)	424 (3.9)	145 (34.2)	10,396 (96.1)	518 (5.0)	< 0.001
Pneumonia/sepsis	11,219 (23.5)	2331 (20.8)	2028 (18.1)	686 (33.8)	9191 (81.9)	1645 (17.9)	< 0.001
Neuro non-stroke	1672 (3.5)	85 (5.1)	304 (18.2)	20 (6.6)	1368 (81.8)	65 (4.8)	0.19
Stroke	1832 (3.8)	597 (32.6)	384 (21.0)	137 (35.7)	1448 (79.0)	460 (31.8)	0.15
Substance abuse/psychosis	2469 (5.2)	153 (6.2)	723 (29.3)	53 (7.3)	1746 (70.7)	100 (5.7)	0.13
Other diagnoses	6495 (13.6)	515 (7.9)	417 (6.4)	116 (27.8)	6078 (93.6)	399 (6.6)	< 0.001

^*^Chi-square test testing for association between death after intubation and death without intubation within each principal diagnosis group

### Predictors of intubation

When we assessed for collinearity, due to our large sample size, though we saw statistically significant results between some factors, the correlations were small and not meaningful*.* Among the 31,053 patients in derivation cohort, the strongest predictors for intubation were presence of additional organ failure (in addition to acute respiratory failure) and principal diagnosis groups. A final model was built in full cohort including these selected 10 factors. Compared to the referent group with principal diagnosis of AMI, patients with stroke had about 6.2 times higher odds of NIV failure and those with a non-stroke neurological conditions, 5.4 times higher odds of intubation. Patients with two or more organ failures in addition to ARF at the time of admission had 5.3 times higher odds of intubation compared to those without additional organ failure. Also, the following factors increased the odds of intubation: prior year IMV use by a factor of 3.0, pneumonia as a comorbid condition with an odds ratio of 2.2, and tachypnea with an odds ratio of 1.9. The model performed well with a c-statistic of 0.81. The main predictors of intubation and the scoring system from the associated model coefficients from full cohort are presented in Table 2.

**Table 2:** Predictors for intubation in derivation and full cohorts

Factor	Derivation cohort	Full cohort	
	Odds ratio (95% CI)	Odds ratio (95% CI)	Points
*Number of organs failed*			
None	Referent	Referent	
One	2.29 (2.08, 2.53)	2.37 (2.19, 2.56)	4
Two or more	5.46 (4.95, 6.02)	5.34 (4.94, 5.78)	8
*Primary diagnostic group*			
Acute myocardial infarct	Referent	Referent	
COPD/asthma	3.24 (2.77, 3.80)	2.94 (2.59, 3.34)	5
Congestive heart failure	1.80 (1.49, 2.17)	1.52 (1.30, 1.78)	2
Pneumonia or Sepsis	3.94 (3.40, 4.56)	3.54 (3.15, 3.98)	6
Neuro non-stroke	5.53 (4.55, 6.72)	5.35 (4.56, 6.32)	8
Stroke	6.84 (5.62, 8.33)	6.18 (5.28, 7.24)	8
Substance abuse or psychosis	10.09 (8.47, 12.01)	8.70 (7.58, 9.99)	10
Other	1.78 (1.49, 2.14)	1.54 (1.33, 1.77)	2
*Pneumonia (secondary dgn)*	2.27 (2.04, 2.53)	2.21 (2.02, 2.42)	4
*Prior year invasive mechanical vent*	2.98 (2.51, 3.53)	3.05 (2.65, 3.51)	5
*Systolic blood pressure*			
≥ 91	Referent	Referent	
≤ 90	1.55 (1.42, 1.68)	1.51 (1.42, 1.62)	2
*Body mass index*			
18.0—25.0	Referent	Referent	
< 18.5	1.18 (1.02, 1.36)	1.23 (1.10, 1.39)	1
*Bicarbonate*			
22 – 27	Referent	Referent	
< 22	0.96 (0.88, 1.04)	1.02 (0.96, 1.10)	1
*Weight loss (secondary dgn)*	1.51 (1.35, 1.68)	1.48 (1.35, 1.62)	2
*No prior year admissions*	1.42 (1.31, 1.55)	1.41 (1.32, 1.51)	2
*Respiratory rate*			
≤ 29	Referent	Referent	
≥ 30	1.75 (1.33, 2.30)	1.92 (1.53, 2.40)	3

For physiological variables we chose the worst value in the 24 h prior to NIV initiation

### Model validation

Characteristics of patients in derivation and validation cohorts are in Additional file 1: Table E2. We observed that compared with the derivation cohort, the validation cohort patients were less likely to be Black, more likely to have AMI, and less likely to have hypercarbia; there were no significant differences in the outcome rate. When the intubation simplified score was applied to the validation set, the c-statistic was 0.80. Predicted and observed intubation rates in the validation cohort were in close agreement except in the highest deciles (Additional file 1: Figure E1 in online data supplement). C-statistics from the fivefold validation showed good discrimination with values of 0.80 or greater across the fivefolds showing robustness of the final model.

### Risk score

**Fig. 2:**
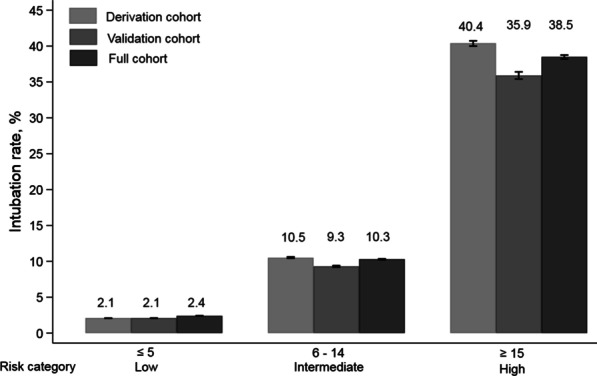
Risk score categories and associated probability of intubation

Ten variables were included in the final risk score: organ failure, principal diagnosis, secondary diagnosis of pneumonia or weight loss, requiring IMV in the prior year, low BMI, presence of tachypnea, presence of hypotension, low bicarbonate, and not having admissions in prior year. A patient who falls into each of the scored variable category can have a maximum score of 38 with a risk of intubation of about 97.9%. Patients who were intubated had a significantly higher risk score (median: 14, IQR: 10–17) than patients who were not intubated (median: 7, IQR: 3–10). The median, interquartile range and overall range of risk of intubation in the 3 risk categories of the scoring system were: 2.3% (IQR: 1.9%–2.8%; range: < 4.0%) for the low risk category (score ≤ 5); 9.3% (IQR: 6.3%–13.5%; range: 4.0%—23%) for intermediate risk category (score 6–14); and 35.7% (IQR: 31.0%–45.8%; range: > 23%) for high risk category (score ≥ 15) (Fig. 2).

Table 3 shows the predictors and the risk score for NIV failure defined as intubation or death. Although the points for individual factors changed, the same variables were included.

**Table 3:** Predictors for noninvasive ventilation failure (intubation or death)

Factor	Intubation or death			Score
	OR	LL	UL	
*Number of organs failed*				
None	Referent			
One	1.646	1.543	1.755	2
Two or more	3.733	3.501	3.981	6
*Primary diagnostic group*				
Acute myocardial infarct	Referent			
COPD/asthma	1.804	1.634	1.991	3
Congestive heart failure	1.275	1.135	1.433	1
Pneumonia or sepsis	3.464	3.181	3.771	5
Neuro non-stroke	2.79	2.423	3.213	5
Stroke	9.664	8.565	10.903	10
Substance abuse or psychosis	4.293	3.828	4.815	6
Other	1.343	1.21	1.491	1
*Pneumonia (secondary dgn)*	1.966	1.818	2.127	3
*Prior year invasive mechanical vent*	1.913	1.682	2.176	3
*Systolic blood pressure*				
≥ 91	Referent			
≤ 90	2.07	1.959	2.187	3
*Body mass index*				
18.0–25.0	Referent			
< 18.5	1.372	1.241	1.517	2
*Bicarbonate*				
22 – 27	Referent			
< 22	0.737	0.697	0.779	1
*Weight loss (secondary dgn)*	1.19	1.097	1.291	1
*Respiratory rate*				
≤ 29	Referent			
≥ 30	2.507	2.064	3.044	4
				c-stat: 0.78

For physiological variables we chose the worst value in the 24 h prior to NIV initiation

## Discussion

Using a large cohort of non-surgical patients treated with NIV at 127 US hospitals, we found that a simple model using data available at hospital presentation successfully predicted intubation after initial treatment with NIV. The final risk score includes number of organ failure, principal diagnosis, acute physiological parameters, and chronic disease comorbidities, and provides a simple method to stratify a patient’s risk of NIV failure into low and high risk categories relative to an intermediate group at average risk. Because of the large size of our cohort and the large network of hospitals contributing data, our model is statistically robust and highly generalizable. This model has significant potential for being incorporated in an online prognostic calculator (see example Additional file 1: figure E2 in the supplement) to help routine decision-making by providers and support appropriate monitoring and/or counseling of patients and families. We have also developed a risk score for NIV failure defined as intubation or death which included the same factors as the intubation only model, although the weight of the predictors changed slightly. Of note, our risk score applies to patients started on NIV soon after admission and not to patients who develop respiratory distress and are treated with NIV later in the course of hospitalization.

The present model differs from prior models used to predict intubation in patients started on NIV in several ways [15, 21–24]. First, our model was designed to be used in any non-surgical patient started on NIV, regardless of the principal diagnosis, allowing for broader utility. Therefore, our study was not restricted to specific conditions such as COPD or CHF where the evidence for use of NIV is strong. Instead, we developed our predictive model in a large group of patients treated with NIV in routine clinical settings. Several predictive scores exist for specific diagnoses. For example, Confalonieri and colleagues developed a prediction chart of failure risk in patients with COPD [22]. They found that patients with an APACHE II score ≥ 29, a Glasgow coma score < 11, and a respiratory rate ≥ 30 breaths/min have a predicted risk of NIV failure of > 70%. However, inclusion of the APACHE II score makes it less practical due to the multiple variables needed, including laboratory tests. Second, our approach is novel in that is using a large EHR dataset. The variables in our model are easily obtainable and the scoring could be applicable not only for clinical purposes but also for studies with administrative data. Third, we have developed a tool to quantitatively estimate the risk for intubation. If the risk is high, clinicians have to make the difficult decision between NIV and IMV given that those who fail NIV have mortality which is similar or even higher than those started on IMV [9, 16]. Prior studies have shown that at least part of the increase in mortality is related to delayed intubation; this is why, if NIV is started in patients at high risk for failure, these patients need to be closely watched in a highly monitored environment. In this study we found that when NIV was started for unusual diagnoses such as drug overdose or seizure, the risk of NIV failure was high; for this group, the decision to intubate has to be seriously considered. Fourth, the prognostic model can be used as an aid in making decisions about placement of patients in ICU or intermediate care, thereby matching the intensity of monitoring with the needs of the patient [36, 37]. Of note, in a step-down unit, patients are generally monitored with the same technology as in an ICU but frequency of monitoring and the intensity of care provided by the nurses and respiratory therapists is lower [38, 39]. Currently, there is large variation in policies regarding administration of NIV across hospitals, with some institutions restricting NIV utilization to the ICU while others allow it on step-down units [20, 37]. Our scoring system can help tailor these decisions. For example, patients with substance abuse, pneumonia, renal failure (one organ dysfunction), cachexia, a prior year intubation, and tachypnea will have a total score of 28, giving them an 85% probability for NIV failure (Additional file 1: Figure E2); consequently, these patients should be closely watched in the ICU or intubated in the first place.

Our results largely confirm a number of risk factors for NIV failure that have been previously described by other studies [9, 11, 15, 17]. However, a surprising finding of our study is the large number of patients treated with NIV who had neurological, substance abuse, or psychiatric diagnoses; most of which are not typical for acute respiratory patients. Notably, only 35.3% of the 5,973 patients with these diagnoses had a secondary diagnosis of conditions that would suggest an indication for NIV such as CHF, asthma, COPD, AMI, pneumonia or sepsis, raising questions on the purpose of using NIV in this cohort. Furthermore, this group had a higher risk for intubation compared with patients with CHF or COPD: almost one in three patients in this category needed to be intubated after a trial of NIV, demonstrating that they are not good candidates for NIV. We are not able to identify the reason why these patients were started on NIV. One could hypothesize that these patients became lethargic and hypo-ventilated due to their primary diagnosis and consequently became hypoxic or hypercarbic, triggering the use of NIV. While it is true that these are not standard indications for NIV, our data reflect routine care in a large unselected population.

Organ failure was a strong predictor for intubation or NIV failure and patients with two or more organ failures were five times more likely to experience failure than those without organ failure. Although there is strong evidence that organ failure is an important risk factor for intubation or NIV failure, in this real-world cohort 35% of patients treated with NIV and 62% of those who failed NIV had at least one organ failure. This scoring system could help providers to be more vigilant when choosing to deliver NIV to a patient with relative contraindications for NIV.

The results of our study should be interpreted considering its limitations. First, we did not have data on clinical assessments at the 1–2 h time point after initiation of NIV, findings that have been shown to predict NIV success [24]. Nevertheless, our model was intended to provide prognostic information at the time of NIV initiation. Evaluating the response to the NIV is a key aspect of management. However, once the follow-up assessment is made with our risk score, one can adjust the initial prediction (aka ‘prior probability’) based on the new information. Second, our outcome was NIV intubation and did not take in account the competing risk of death (8.9% of patients died without being intubated). For this reason, we have also developed a predictive score for intubation or death. Third, we relied on ICD-9CM and ICD-10CM diagnostic codes which could have resulted in misclassification. Fourth, we lacked information on advance directive status and therefore patients with a do-not-intubate status could have been retained in the cohort. Fifth although the prediction model was validated via a temporal external cohort, future validation in another cohort including additional sites is needed. Sixth, we did not have information about the use of high flow nasal oxygen in this population. Finally, this model does not apply to surgical patients or those with OSA who were excluded from the cohort.

## Conclusions

Clinical variables at the time of admission can be used to accurately predict the risk of intubation and of intubation or death, in a broad sample of hospitalized patients using readily available clinical data. The prognostic score may provide quantitative guidance for decision-making about patients with acute respiratory failure who may require conventional mechanical ventilation. Although multiple risk scores for intubation or NIV failure exist few are utilized in routine care. Our score which is applicable to any adult patient for whom a provider is considering NIV could be built in a web-based calculator for easy use at the point-of-care.

## Supplementary Information

**Additional file 1: Figure E1:** Observed vs. Predicted intubation in derivation and validation cohorts. **Figure E2:** Example of total score and risk of intubation for patients satisfying certain conditions from the model. **Table E1:** Characteristics of patients with and without intubation. ^§^ Conditions identified as organ failure include: cardiovascular failure/shock, renal failure, neurological failure, hematological failure, hepatic failure, acidosis *measured on same day as NIV initiation (days 0 or 1 or 2). †Kruskal–Wallis test. #Chi-square test. ^Missing values among patients: 9081 (19%) for BMI; 929 (1.9%) for respiratory rate; 2551 (5.3%) for systolic blood pressure. These were grouped as separate categories and included in all analyses. **Table E2:** Characteristics of patients in derivation and validation cohorts for intubation outcome. ^§^ Conditions identified as organ failure include: cardiovascular failure/shock, renal failure, neurological failure, hematological failure, hepatic failure, acidosis. *measured on same day as NIV initiation (days 0 or 1 or 2). †Kruskal–Wallis test. #Chi-square test. ^Missing values among patients: 9081 (19%) for BMI; 929 (1.9%) for respiratory rate; 2551 (5.3%) for systolic blood pressure. These were grouped as separate categories and included in all analyses.

## Data Availability

The dataset are not publicly available due to the data agreement with Cerner/HealthFacts Company not to share the data of the hospitals contributing to the dataset. The source of data is Cerner HealthFacts https://sc-ctsi.org/resources/cerner-health-facts.
