# Supplementary material for: A scoring system derived from electronic health records to identify patients at high risk for noninvasive ventilation failure
Source: BMC Pulm Med. 2021 Feb 5;21:52. doi: 10.1186/s12890-021-01421-w (PMC7863252; doi:10.1186/s12890-021-01421-w)
Supplement: Supplementary file 1 — Additional file 1: Figure E1: Observed vs. Predicted intubation in derivation and validation cohorts. Figure E2: Example of total score and risk of intubation for patients satisfying certain conditions from the model. Table E1: Characteristics of patients with and without intubation. § Conditions identified as organ failure include: cardiovascular failure/shock, renal failure, neurological failure, hematological failure, hepatic failure, acidosis *measured on same day as NIV initiation (days 0 or 1 or 2). †Kruskal–Wallis test. #Chi-square test. ^Missing values among patients: 9081 (19%) for BMI; 929 (1.9%) for respiratory rate; 2551 (5.3%) for systolic blood pressure. These were grouped as separate categories and included in all analyses. Table E2: Characteristics of patients in derivation and validation cohorts for intubation outcome. § Conditions identified as organ failure include: cardiovascular failure/shock, renal failure, neurological failure, hematological failure, hepatic failure, acidosis. *measured on same day as NIV initiation (days 0 or 1 or 2). †Kruskal–Wallis test. #Chi-square test. ^Missing values among patients: 9081 (19%) for BMI; 929 (1.9%) for respiratory rate; 2551 (5.3%) for systolic blood pressure. These were grouped as separate categories and included in all analyses. [file 12890_2021_1421_MOESM1_ESM.docx]

**Supplemental material**

**A Scoring System Derived from Electronic Health Records to Identify Patients at High Risk for Noninvasive Ventilation Failure**

Mihaela S Stefan MD, PhD^1,2^; Aruna Priya MA, MSc^1^, Penelope S Pekow PhD^1,3^, Jay Steingrub MD^4^, Nicholas S Hill MD^5^, Tara Lagu MD MPH^1,2^, Karthik Raghunathan MD, MPH^6^, Anusha G Bhat MD^2^, Peter K Lindenauer MD, MSc^1,2,7^

^1^Institute for Healthcare Delivery and Population Science, University of Massachusetts Medical School - Baystate, Springfield, MA

^2^Department of Medicine, University of Massachusetts Medical School - Baystate, Springfield, MA

^3^School of Public Health and Health Sciences, University of Massachusetts, Amherst, MA

^4^ Division of Pulmonary and Critical Care, Department of Medicine, University of Massachusetts Medical School - Baystate, Springfield, MA

^5^Division of Pulmonary and Critical Care Tufts University School of Medicine, Boston, MA

^6^Division of Veterans Affairs, Department of Anesthesiology, Duke University Medical Center, Durham, NC

^7^Department of Quantitative Health Sciences, University of Massachusetts Medical School, Worcester, MA

**Figure E1:** Observed vs. Predicted intubation rates in derivation and validation cohorts


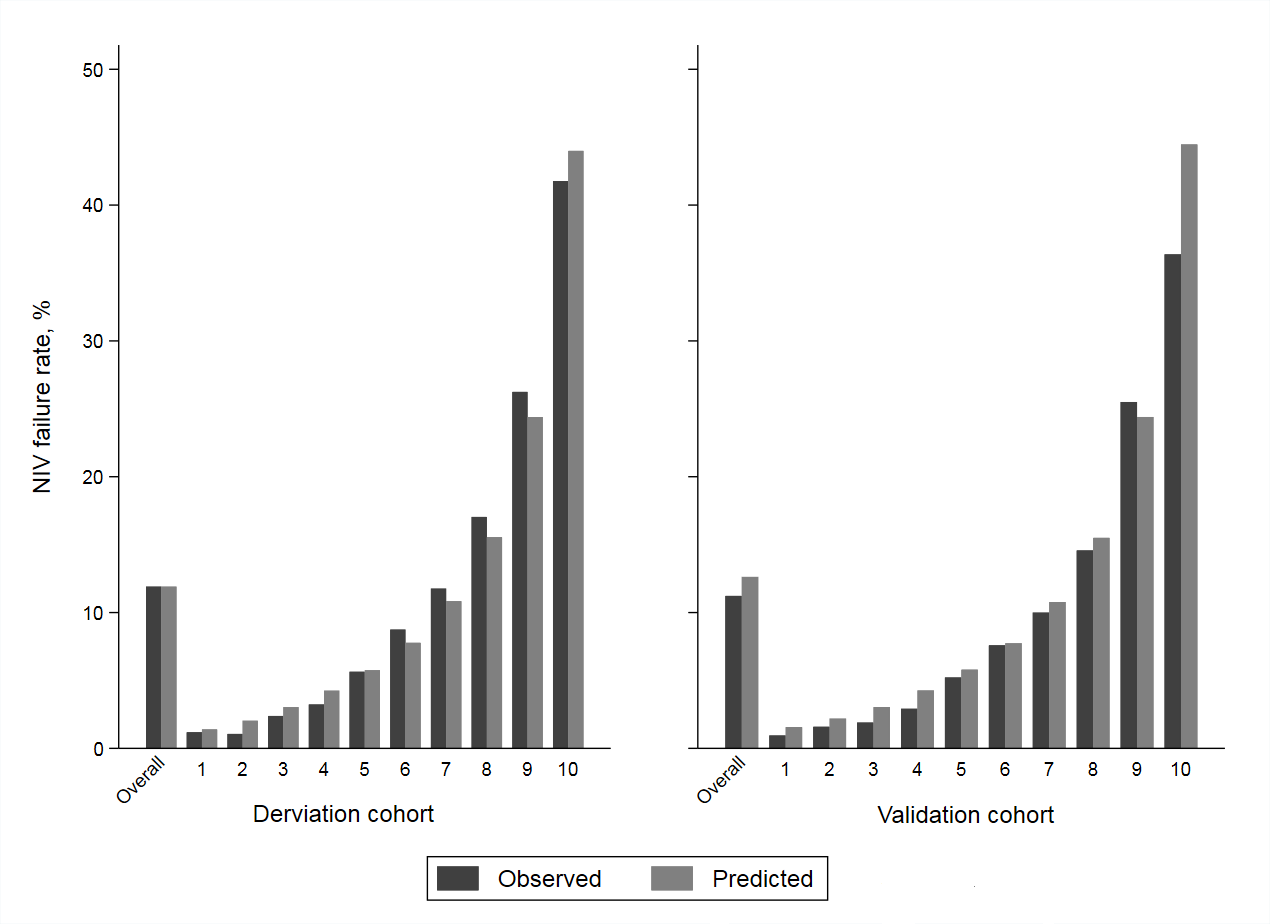


**Figure E2:** Example of total score and risk of intubation for patients satisfying certain conditions from the model


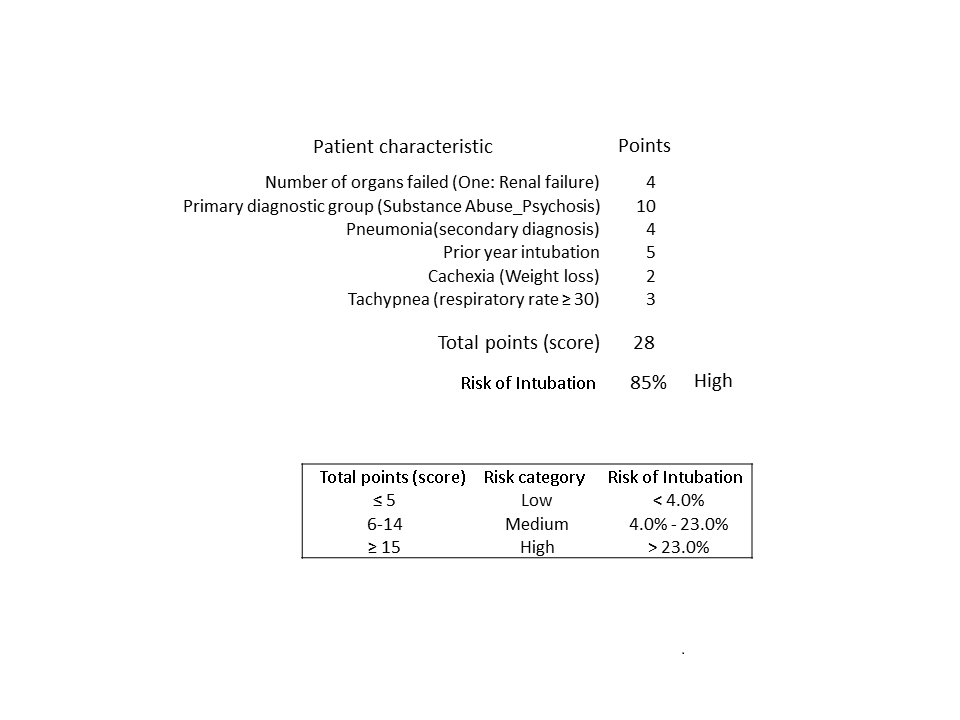


**Table E1:** Characteristics of patients with and without intubation

| **Characteristics** | **Total** | **No NIV failure** | **NIV failure** | **Absolute standardized difference** |
| --- | --- | --- | --- | --- |
|  | **N (%)** | **n (%)** | **n (%)** | **%** |
|  | **47,749 (100)** | **42177 (88.3)** | **5,572 (11.7)** |  |
| **Age, Median (IQR), years** | 65 (54 - 76) | 66 (55 - 76) | 62 (50 - 73) | 24.1 |
| **Female** | 23022 (48.2) | 20312 (48.2) | 2710 (48.6) | 0.9 |
| **Race/Ethnicity** |  |  |  | 18.4 |
| White | 35723 (74.8) | 31940 (75.7) | 3783 (67.9) |  |
| Black | 7700 (16.1) | 6475 (15.4) | 1225 (22) |  |
| Hispanic | 521 (1.1) | 449 (1.1) | 72 (1.3) |  |
| Other | 3805 (8) | 3313 (7.9) | 492 (8.8) |  |
| **Principal diagnosis groups** |  |  |  | 70.9 |
| COPD/ASTHMA | 8346 (17.5) | 7405 (17.6) | 941 (16.9) |  |
| Congestive heart failure | 4896 (10.3) | 4545 (10.8) | 351 (6.3) |  |
| Acute myocardial infact | 10820 (22.7) | 10396 (24.7) | 424 (7.6) |  |
| Pneumonia/Sepsis | 11219 (23.5) | 9191 (21.8) | 2028 (36.4) |  |
| Neuro Non-stroke | 1672 (3.5) | 1368 (3.2) | 304 (5.5) |  |
| Stroke | 1832 (3.8) | 1448 (3.4) | 384 (6.9) |  |
| Substance abuse/Psychosis | 2469 (5.2) | 1746 (4.1) | 723 (13.0) |  |
| Other diagnoses | 6495 (13.6) | 6078 (14.4) | 417 (7.5) |  |
|  |  |  |  |  |
| **Comorbidities (secondary diagnoses)** |  |  |  |  |
| Neurological disorders | 5699 (11.9) | 4479 (10.6) | 1220 (21.9) | 30.9 |
| Renal failure | 7730 (16.2) | 6659 (15.8) | 1071 (19.2) | 9 |
| Liver disease | 1435 (3.0) | 1083 (2.6) | 352 (6.3) | 18.3 |
| Malignancy | 2028 (4.2) | 1710 (4.1) | 318 (5.7) | 7.7 |
| Obesity | 6364 (13.3) | 5555 (13.2) | 809 (14.5) | 3.9 |
| Weight loss | 3891 (8.1) | 2925 (6.9) | 966 (17.3) | 32.3 |
| Congestive heart failure | 9152 (19.2) | 7802 (18.5) | 1350 (24.2) | 14 |
| COPD | 10257 (21.5) | 9036 (21.4) | 1221 (21.9) | 1.2 |
| Psychiatric/Substance abuse | 4963 (10.4) | 4009 (9.5) | 954 (17.1) | 22.6 |
| Pneumonia | 8686 (18.2) | 6498 (15.4) | 2188 (39.3) | 55.6 |
| **Number of organs failed^§^ (count)** |  |  |  | 85.6 |
| No organ failure | 30230 (63.3) | 28505 (67.6) | 1725 (31.0) |  |
| Failure of 1 organ | 9569 (20.0) | 8160 (19.3) | 1409 (25.3) |  |
| Failure of 2 or more organs | 7950 (16.6) | 5512 (13.1) | 2438 (43.7) |  |
| **Vasopressors** | 7810 (16.4) | 6407 (15.2) | 1403 (25.2) | 25.1 |
| **Prior year hospitalizations** |  |  |  | 8.9 |
| 0 (no admits) | 28628 (60.0) | 25074 (59.4) | 3554 (63.8) |  |
| 1 admit | 9383 (19.6) | 8395 (19.9) | 988 (17.7) |  |
| 2 or more admits | 9738 (20.4) | 8708 (20.6) | 1030 (18.5) |  |
| **Prior year Invasive mechanical vent.** |  |  |  | 19.6 |
| 0 | 46250 (96.9) | 41057 (97.3) | 5193 (93.2) |  |
| 1 time | 308 (0.6) | 233 (0.6) | 75 (1.3) |  |
| 2 or more times | 1191 (2.5) | 887 (2.1) | 304 (5.5) |  |
| **Prior year Noninvasive ventilation** |  |  |  | 10 |
| 0 | 42263 (88.5) | 37294 (88.4) | 4969 (89.2) |  |
| 1 time | 1047 (2.2) | 988 (2.3) | 59 (1.1) |  |
| 2 or more times | 4439 (9.3) | 3895 (9.2) | 544 (9.8) |  |
| **Laboratory/Vitals** |  |  |  |  |
| PaO2*, Median (IQR) | 85 (65 - 129) | 85 (65 - 128) | 86.8 (64 - 136.7) | 5.9 |
| PaCo2*, Median (IQR) | 38.2 (32.3 - 47) | 38.7 (33 - 48) | 37 (31 - 44) | 18.7 |
| Ph*, Median (IQR) | 7.3 (7.2 - 7.4) | 7.3 (7.26 - 7.39) | 7.3 (7.2 - 7.4) | 14.1 |
| **BMI*^** |  |  |  | 27.3 |
| <18.5 | 2853 (6.0) | 2304 (5.5) | 549 (9.9) |  |
| 18.5 - 25 | 13119 (27.5) | 11184 (26.5) | 1935 (34.7) |  |
| >25 | 22696 (47.5) | 20475 (48.6) | 2221 (40.0) |  |
| **Respiratory rate^** |  |  |  | 11.3 |
| <=29 | 46263 (96.9) | 40882 (96.9) | 5381 (96.6) |  |
| >=30 | 557 (1.2) | 434 (1.0) | 123 (2.2) |  |
| **Systolic blood pressure^** |  |  |  | 36.8 |
| <=90 | 12264 (25.7) | 10002 (23.7) | 2262 (40.6) |  |
| >=91 | 32934 (69.0) | 29885 (70.9) | 3049 (54.7) |  |
| **BUN*** |  |  |  | 11 |
| < 18 | 27986 (58.6) | 24846 (58.9) | 3140 (56.4) |  |
| 18 - <40 | 13915 (29.1) | 12352 (29.3) | 1563 (28.1) |  |
| >=40 | 5848 (12.3) | 4979 (11.8) | 869 (15.6) |  |
| **Bicarbonate*** |  |  |  | 27.2 |
| 22 - 27 | 24860 (52.1) | 22207 (52.7) | 2653 (47.6) |  |
| < 22 | 15122 (31.7) | 12789 (30.3) | 2333 (41.9) |  |
| >=28 | 7767 (16.3) | 7181 (17.0) | 586 (10.5) |  |
|  |  |  |  |  |
| **Outcomes** |  |  |  | p-value |
| **LOS, Median (IQR) days^†^** | 5 (3 - 9) | 5 (2 - 8) | 8 (3 - 16) | <0.001 |
| **Mortality^#^** | 5150 (10.8) | 3748 (8.9) | 1402 (25.2) | <0.001 |

**^§^** Conditions identified as organ failure include: cardiovascular failure/shock, renal failure, neurological failure, hematological failure, hepatic failure, acidosis

*measured on same day as NIV initiation (days 0 or 1 or 2)

†Kruskal-Wallis test

#Chi-square test

^Missing values among patients: 9081 (19%) for BMI; 929 (1.9%) for respiratory rate; 2551 (5.3%) for systolic blood pressure. These were grouped as separate categories and included in all analyses.

**Table E2:** Characteristics of patients in derivation and validation cohorts for intubation outcome

| **Characteristics** | **Total** | **Validation** | **Derivation** | **Absolute standardized difference** |
| --- | --- | --- | --- | --- |
|  | **N (%)** | **n (%)** | **n (%)** | **%** |
|  | **47,749 (100)** | **16,696 (35)** | **31,053 (65)** |  |
| **Age, Median (IQR), years** | 65 (54 - 76) | 65 (54 - 75) | 65 (54 - 76) | 3.59 |
| **Female** | 23022 (48.2) | 7905 (47.4) | 15117 (48.7) | 2.7 |
| **Race/Ethnicity** |  |  |  | 10.3 |
| White | 35723 (74.8) | 12396 (74.3) | 23327 (75.1) |  |
| Black | 7700 (16.1) | 2927 (17.5) | 4773 (15.4) |  |
| Hispanic | 521 (1.1) | 115 (0.7) | 406 (1.3) |  |
| Other | 3805 (8) | 1258 (7.5) | 2547 (8.2) |  |
| **Principal diagnosis groups** |  |  |  | 12.6 |
| COPD/ASTHMA | 8346 (17.5) | 2825 (16.9) | 5521 (17.8) |  |
| CHF | 4896 (10.3) | 1682 (10.1) | 3214 (10.4) |  |
| AMI | 10820 (22.7) | 3282 (20.0) | 7538 (24.3) |  |
| Pneumonia/Sepsis | 11219 (23.5) | 4030 (24.1) | 7189 (23.2) |  |
| Neuro Non-stroke | 1672 (3.5) | 651 (3.9) | 1021 (3.3) |  |
| Stroke | 1832 (3.8) | 670 (4.0) | 1162 (3.7) |  |
| Substance abuse/Psychosis | 2469 (5.2) | 961 (5.8) | 1508 (4.9) |  |
| Other diagnoses | 6495 (13.6) | 2595 (15.5) | 3900 (12.6) |  |
|  |  |  |  |  |
| **Comorbidities (secondary diagnoses)** |  |  |  |  |
| Neurological disorders | 5699 (11.9) | 1911 (11.5) | 3788 (12.2) | 2.3 |
| Renal failure | 7730 (16.2) | 2462 (14.8) | 5268 (17.0) | 6.1 |
| Liver disease | 1435 (3.0) | 480 (2.9) | 955 (3.1) | 1.2 |
| Malignancy | 2028 (4.2) | 597 (3.6) | 1431 (4.6) | 5.2 |
| Obesity | 6364 (13.3) | 2113 (12.7) | 4251 (13.7) | 3.1 |
| Weight loss | 3891 (8.1) | 1329 (8.0) | 2562 (8.3) | 1.1 |
| CHF | 9152 (19.2) | 2819 (16.9) | 6333 (20.4) | 9 |
| COPD | 10257 (21.5) | 3360 (20.1) | 6897 (22.2) | 5.1 |
| Psychiatric/Substance abuse | 4963 (10.4) | 1640 (9.8) | 3323 (10.7) | 2.9 |
| Pneumonia | 8686 (18.2) | 2918 (17.5) | 5768 (18.6) | 2.9 |
| **Number of organs failed (count) ^§^** |  |  |  | 3.3 |
| No organ failure | 30230 (63.3) | 10589 (63.4) | 19641 (63.3) |  |
| Failure of 1 organ | 9569 (20.0) | 3206 (19.2) | 6363 (20.5) |  |
| Failure of 2 or more organs | 7950 (16.6) | 2901 (17.4) | 5049 (16.3) |  |
| **Vasopressors** | 7810 (16.4) | 2033 (12.2) | 5777 (18.6) | 17.9 |
| **Prior year hospitalizations** |  |  |  | 5.2 |
| 0 (no admits) | 28628 (60.0) | 10225 (61.2) | 18403 (59.3) |  |
| 1 admit | 9383 (19.6) | 3299 (19.8) | 6084 (19.6) |  |
| 2 or more admits | 9738 (20.4) | 3172 (19.0) | 6566 (21.1) |  |
| **Prior year IMV** |  |  |  | 0 |
| 0 | 46250 (96.9) | 16204 (97.1) | 30046 (96.8) |  |
| 1 time | 308 (0.6) | 115 (0.7) | 193 (0.6) |  |
| 2 or more times | 1191 (2.5) | 377 (2.3) | 814 (2.6) |  |
| **Prior year NIV** |  |  |  | 0 |
| 0 | 42263 (88.5) | 14779 (88.5) | 27484 (88.5) |  |
| 1 time | 1047 (2.2) | 305 (1.8) | 742 (2.4) |  |
| 2 or more times | 4439 (9.3) | 1612 (9.7) | 2827 (9.1) |  |
|  |  |  |  |  |
| **Laboratory/Vitals** |  |  |  |  |
| LAPS, Median (IQR) | 43 (26 - 63) | 43 (25 - 64) | 43 (26 - 63) | 3 |
| PaO2*, Median (IQR) | 85 (65 - 129) | 88 (66.3 - 136.0) | 83 (63.5 - 125) | 5.5 |
| PaCo2*, Median (IQR) | 38.2 (32.3 - 47) | 38 (32 - 46) | 39 (33 - 48) | 9.6 |
| Ph*, Median (IQR) | 7.3 (7.2 - 7.4) | 7.3 (7.2 - 7.4) | 7.33 (7.26 - 7.39) | 2.9 |
| **BMI***^ |  |  |  | 5.2 |
| <18.5 | 2853 (6.0) | 946 (5.7) | 1907 (6.1) |  |
| 18.5 - 25 | 13119 (27.5) | 4550 (27.3) | 8569 (27.6) |  |
| >25 | 22696 (47.5) | 7788 (46.7) | 14908 (48.0) |  |
| **Respiratory rate**^ |  |  |  | 14.3 |
| <=29 | 46263 (96.9) | 16421 (98.4) | 29842 (96.1) |  |
| >=30 | 557 (1.2) | 161 (1.0) | 396 (1.3) |  |
| **Systolic blood pressure**^ |  |  |  | 9.8 |
| <=90 | 12264 (25.7) | 4522 (27.1) | 7742 (24.9) |  |
| >=91 | 32934 (69.0) | 11558 (69.2) | 21376 (68.8) |  |
| **BUN*** |  |  |  | 6.1 |
| < 18 | 27986 (58.6) | 10124 (60.6) | 17862 (57.5) |  |
| 18 - <40 | 13915 (29.1) | 4635 (27.8) | 9280 (29.9) |  |
| >=40 | 5848 (12.3) | 1937 (11.6) | 3911 (12.6) |  |
| **Bicarbonate*** |  |  |  | 12.3 |
| 22 - 27 | 24860 (52.1) | 8736 (52.3) | 16124 (51.9) |  |
| < 22 | 15122 (31.7) | 5697 (34.1) | 9425 (30.4) |  |
| >=28 | 7767 (16.3) | 2263 (13.6) | 5504 (17.7) |  |
|  |  |  |  |  |
| **Outcomes** |  |  |  | p-value |
| **NIV failure^#^** | 5572 (11.7) | 1874 (11.2) | 3698 (11.9) | 0.03 |
| **LOS, Median (IQR) days^†^** | 5 (3 - 9) | 5 (3 - 9) | 5 (2 - 9) | <0.001 |
| **Mortality^#^** | 5150 (10.8) | 1785 (10.7) | 3365 (10.8) | 0.63 |

**^§^** Conditions identified as organ failure include: cardiovascular failure/shock, renal failure, neurological failure, hematological failure, hepatic failure, acidosis

*measured on same day as NIV initiation (days 0 or 1 or 2)

†Kruskal-Wallis test

#Chi-square test

^Missing values among patients: 9081 (19%) for BMI; 929 (1.9%) for respiratory rate; 2551 (5.3%) for systolic blood pressure. These were grouped as separate categories and included in all analyses.
